# Anatomical Study of Arachnoid Granulation in Superior Sagittal Sinus Correlated to Growth Patterns of Meningiomas

**DOI:** 10.3389/fonc.2022.848851

**Published:** 2022-03-30

**Authors:** Yuanliang Ye, Wen Gao, Weilin Xu, Jiangu Gong, Minxing Qiu, Lang Long, Jiuyang Ding

**Affiliations:** ^1^ Department of Neurosurgery, Liuzhou People’s Hospital, Liuzhou, China; ^2^ Department of Neurology, Liuzhou People’s Hospital, Liuzhou, China; ^3^ Department of Neurosurgery, Second Affiliated Hospital, School of Medicine, Zhejiang University, Hangzhou, China; ^4^ Department of Anatomy, Guangxi Medical University, Nanning, China; ^5^ Department of Pathology, Southern Medical University, Guangzhou, China; ^6^ School of Forensic Medicine, Guizhou Medical University, Guiyang, China

**Keywords:** parasagittal meningioma, arachnoid granulations, endoscopy, chordae willisii, superior sagittal sinus

## Abstract

Meningiomas in the parasagittal region were formed by arachnoidal cells disseminated among arachnoid granulations. The purpose of this study was to characterize the morphology of chordae willisii, and AGs found in the superior sagittal sinus. This study used 20 anatomical specimens. Rigid endoscopes were introduced *via* torcula herophili into the sinus lumen. The morphological features of arachnoid granulation and chordae willisii were analyzed, and then arachnoid granulations and chordae willisii were assessed by elastic fiber stains, Masson’s stains, and imaging analysis. Three types of arachnoid granulations were present in the examined sinuses. There were 365 counts of arachnoid granulations in examined sinuses by imaging analysis, averaging 1.36 ± 2.58 per sinus. Types I, II, and III made up 20.27, 45.20, and 34.52% of 268 patients, respectively. Microscopy of chordae willisii transverse sections indicated the existence of a single layer and a multiple-layered dura sinus wall. The dural sinus wall was the thickest one in the superior sagittal sinus. The thickness of longitudinal lamellae was significantly greater than trabeculae. This study reveals the anatomical differences between arachnoid granulations in the superior sagittal sinus. The arachnoid granulations classification enables surgeons to predict preoperatively growth patterns, followed by safely achieving the optimal range of parasagittal meningioma resection.

## Introduction

The parasagittal meningioma (PSM) subgroup comprises 19.5 to 45% of all intracranial meningiomas (1–3). Patients with symptoms are generally treated surgically as there are no effective medical therapies (4, 5). The tendency for these tumors to invade or even encase the superior sagittal sinus (SSS) requires a multimodal treatment approach to reduce the rate of surgical complications (5–7). The PSM was derived from arachnoidal cap cells distributed in the arachnoid granulations (AGs) (8). The AG essentially consisted of four components: a central core, cap cell cluster, arachnoid cell layer, and fibrous capsule (9, 10). The AG included a network of arachnoid cells and connective tissue fibers. (11, 12). AG occurs in the subarachnoid space along the arachnoid membrane, extending into the dural venous sinuses (13, 14) and results in different growth patterns of parasagittal meningioma. However, the relationship between AGs and development of parasagittal meningioma has not been established.

Based on the degree of sinus invasion by imaging analysis, PSM has been classified as various types, aiming to choose the best surgical strategy (15, 16). The membranous structure has been recognized as an effective barrier limiting the extension of the tumors (16, 17). The internal membranous structures in the SSS, especially for chordae willisii (CW), including the different types (bands, bridges, chords, lamellar, trabecular, and valve-like lamellae), were visualized and described as they behaved physiologically with the aid of an endoscope (18, 19). Nevertheless, CWs around AGs, which could affect the sinus extension in meningioma, had not been fully characterized.

We used an advanced rigid endoscope in this study to physiologically describe the distribution of AGs and paid attention to CWs in the SSS. Furthermore, we investigated for arachnoid cell and membranous structure in AG by H&E staining, Masson’s staining, and elastic fiber staining, aiming to illuminate possible growth patterns of parasagittal meningioma.

## Materials and Methods

### Subjects

At the Guangxi Medical University’s Department of Anatomy, 20 anatomical specimens taken during the fresh autopsy were maintained in 10% formalin solution for at least two weeks. Each specimen was over the age of 18 years. This study was approved by the ethics committee of Guangxi Medical University (ID No. KY-2021-007). The following exclusion criteria were used: 1) craniocerebral trauma, 2) neurological illness, and 3) sinus disease. The members of the families signed individual consent permitting the use of resected samples for research.

### Endoscope Assessment

There were 12 male and 8 female specimens with the mean age at death of 62 ± 10.33 years (range: 45–80 years). To describe the intraluminal structure in the SSS, the latex was not injected into vein vessels and sinus. The scalps were removed, and by using a surgical power device (Xishan, China), the cranial vault above the axial plane across the nasion and inion was removed. A 4.5-gauge needle was inserted into the SSS, flushing with tap water to remove blood clots. With the cadavers in supine, fixed in Mayfield head holder, an advanced rigid endoscope (Karl Storz, Germany) with a diameter of 4.0 mm and optics of 0 and 30° was inserted into the sinus lumen from the forehead to the coronal. The endoscopes were connected to a digital camera and a video system, enabling photographic recording of the relevant structures. The morphology of the arachnoid granulation and chordae willisii received special attention. Afterward, the SSS samples were carefully removed en bloc using a surgical microscope (OPMI6, Zeiss), and the SSS samples were cut into 1 cm sections from the torcula herophili. AG and its surrounding structures were placed in the observatory area of interest.

### Light Microscopy Assessment

Following sectioning, the arachnoid granules and their surrounding structures were prepared for microscopic assessment. To assess those morphological characteristics, H&E staining was used in addition to the particular staining method for detecting collagen fibers (Masson’s trichrome) and elastic fibers (Victoria blue). A Zeiss Axioskop plus microscope (Carl Zeiss Microscopy) was used to analyze and document the histological sections at ×50, ×100, and ×400 magnification. Axio Vision software was used to capture and save the images.

### MRI-T_2_WI Analysis

The research involved 268 patients: 167 men and 191 women. At diagnosis, the mean age was 51.63 ± 12.23 years (range: 34–78 years). In addition to conventional cerebral MR sequences, all of these patients acquired 3D high-resolution volumetric MR images [3D T2-SPACE sequence]. Our institutional review board granted approval for this study. This retrospective examination of medical data and imaging studies did not need written informed permission. Consensual analysis of all MR images was performed by two neuroradiologists. Arachnoid granulation was hyperintense on T2WI, and CWs were isointense. Each case was carefully evaluated to determine the numbers and location. Exclusion criteria included the following: (1) cerebral vascular diseases involved with SSS; (2) intracranial tumor involved with SSS; and (3) image data were incomplete or of poor image quality. As previously described, MRI images were captured (14, 20).

### Statistical Analysis

SPSS 22.0 for Windows was used to perform all statistical analyses (SPSS Inc., Chicago, Illinois). Descriptive statistics was used to summarize the categorical data, such as arachnoid granulations and percentages. Means, standard deviations, minimums, and maximums were used to express numerical data. Pearson’s chi-square tests were used to determine any statistical difference about proportions. Continuous variables were compared using independent t-test.

## Results

### Arachnoid Granulation

#### Endoscopic Observations

**Figure 1:**
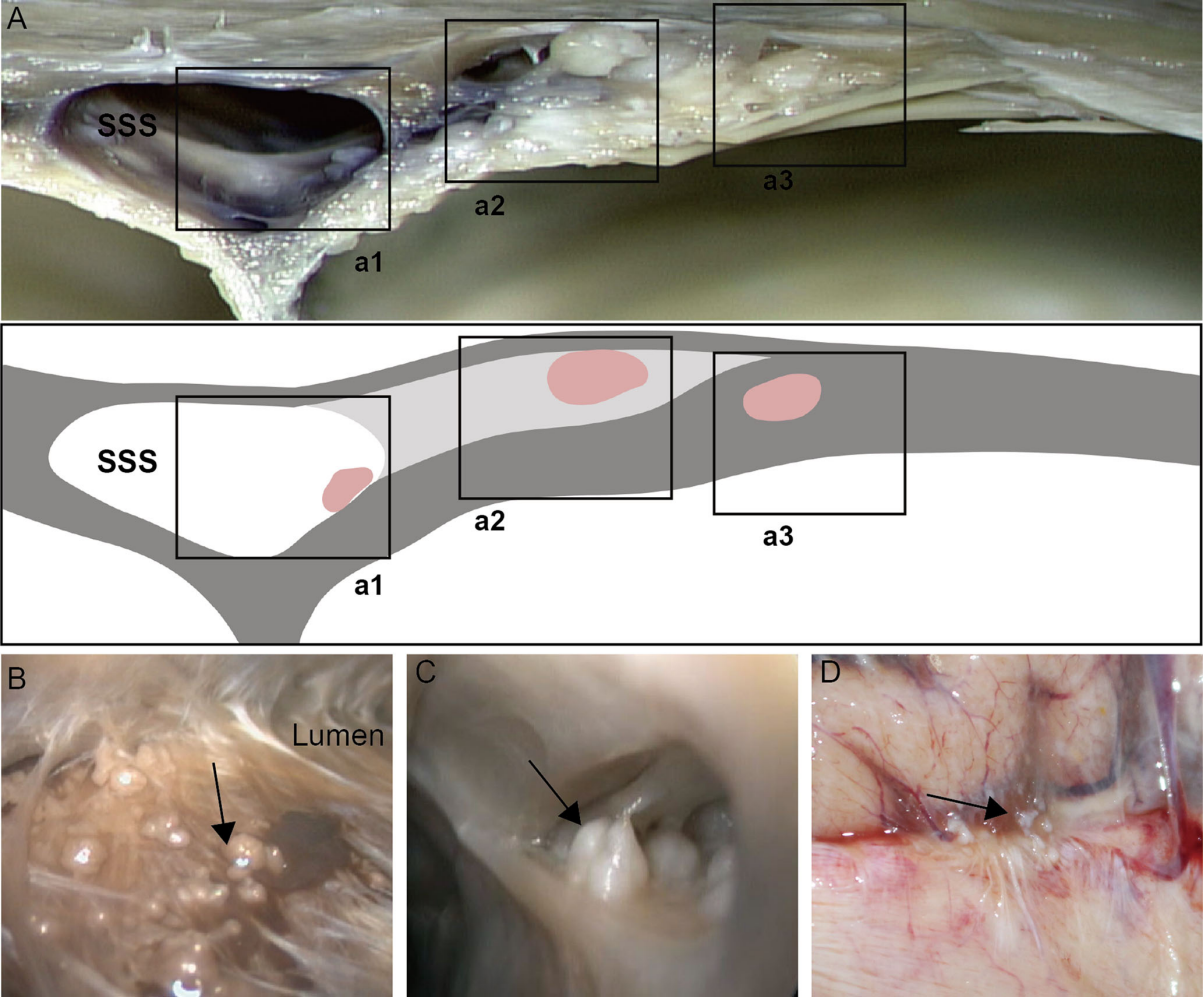
Distribution of arachnoid granulation (black arrow) in cerebral venous sinus. Arachnoid granulation in the lumen **(a1, B)**, lateral sinus **(a2, C)** and subarachnoid space **(a3, D)**.

Various sizes of AG were presented either single or in a cluster. The endoscopic study showed AGs with different distributions physiologically that we classified in three types based on their location (**Figure 1**). The first type (type I): the arachnoid granulations were fixed on the lateral wall of the sagittal sinus and faced to the lumen directly (**Figure 1a1**). The second type (type II): AGs were located in the chambers formed by CW and sinus walls; the surface of these AGs was covered with a transparent membrane (**Figure 1a2**). The third type (type III): AGs were demonstrated in the junction between the side and upper walls and protruded into subarachnoid space around the sagittal sinus, attached to an arachnoid tightly (**Figure 1a3**).

#### Morphological Observations

**Figure 2:**
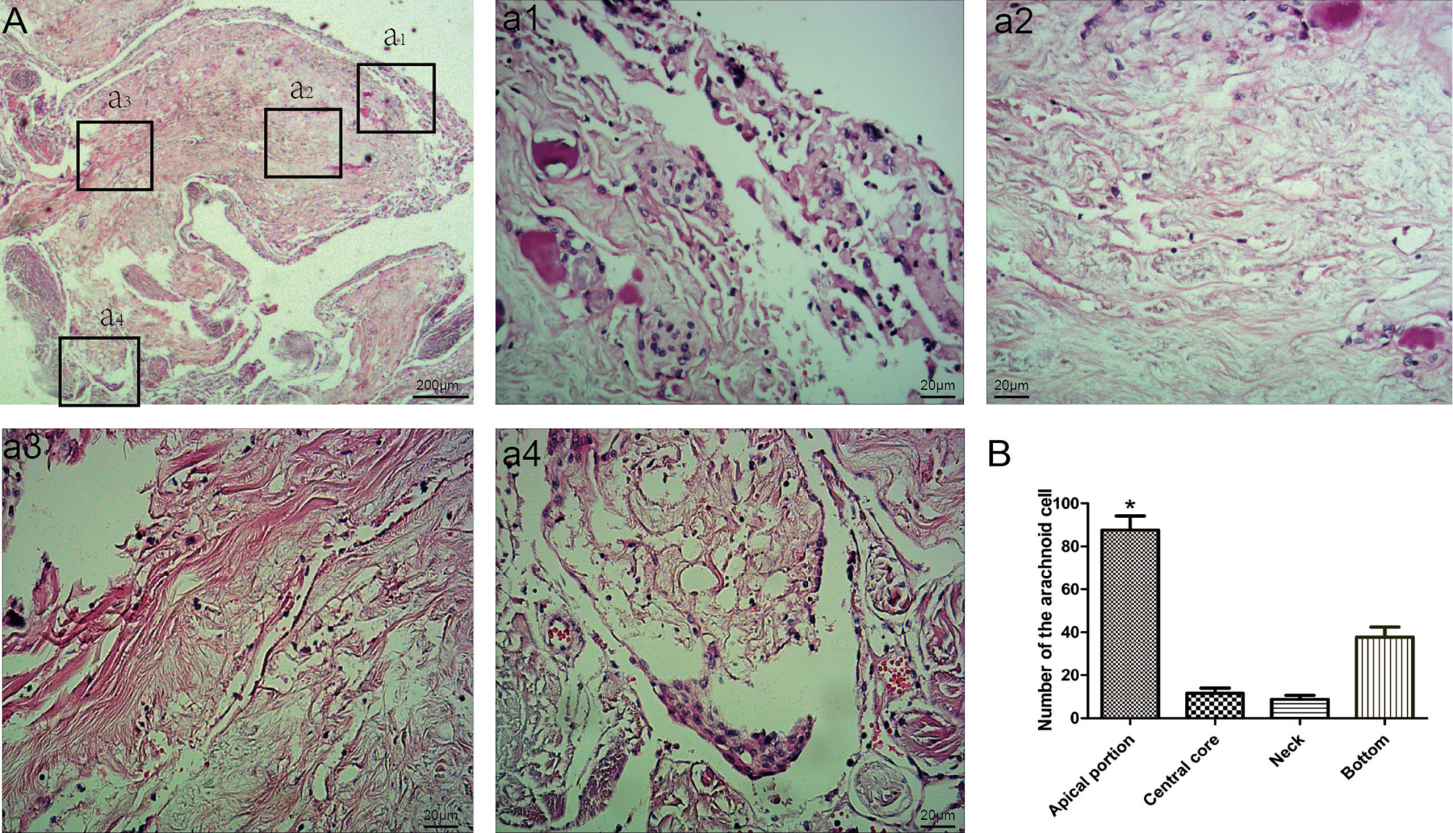
Morphological characteristics of arachnoid granulations. Endothelial cells and arachnoid cells present in the cap cell section (**a1**; HE staining, ×200); Loose connective tissue can be seen in the central core, with a net-like structure (**a2**; HE staining, ×200); Dense connective tissue at the base (**a3**; HE staining, ×200); A large number of vacuole-like tissues present in the neck (**a4**; HE stain, ×200); Comparison of the number of arachnoid cells in different parts; *: means compared with central core,neck and bottom, (P < 0.05) **(B)**.

The arachnoid cell layer encompassing the central core was covered by a fibrous capsule with an endothelial investment. A large number of vacuole-like tissues were present in the neck. The number of arachnoid cells was more in the apical portion compared with that in the central core (**Table 1** and **Figure 2**). The collagen fibers in the junction between AG and the side wall were arranged irregularly. Type I AG has the largest diameter and type II AG has the smallest diameter (**Table 2**).

**Table 1:** Characteristics of AG, CW and dural wall in the superior sagittal sinus.

Arachnoid cell number in AG subregion	Mean ± SD
Apical portion	87.6 ± 6.58
Central core	11.6 ± 2.41
Neck	8.80 ± 1.92
Bottom	37.80 ± 4.66
CW thickness around AG	mm (Mean ± SD)
trabeculae	0.26 ± 0.19
Valve-like lamellae	0.42 ± 0.36
Longitudinal lamellae	0.73 ± 0.51
Dural wall thickness	mm (Mean ± SD)
Side wall	0.82 ± 0.48
Upper wall	0.92 ± 0.39

**Table 2:** Type of AG in cadaveric specimens and 268 patients.

AG Type	AGs by endoscopy	AGs by imaging	AGs by morphological (mean ± SD, mm)
	No. (%)	No. (%)	
Type I	33 (16.58)	74 (20.27)	0.75 ± 0.36
Type II	91 (45.73)	165 (45.20)	0.31 ± 0.28
Type III	75 (37.68)	126 (34.52)	0.52 ± 0.26

#### Imaging Analysis

**Figure 3:**
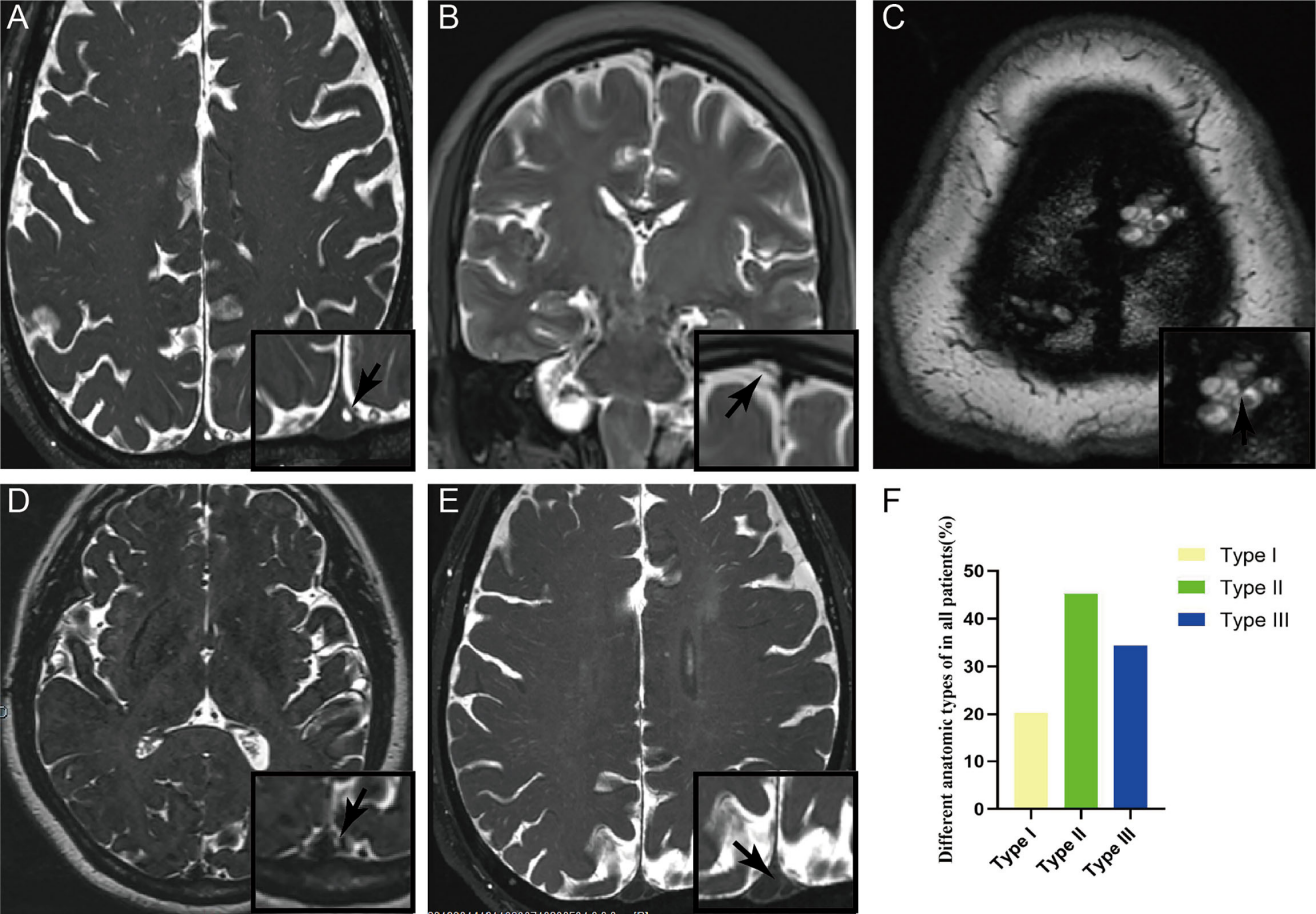
Three types of arachnoid granulation in the superior sagittal sinus were delineated from normal cerebral tissues with hyper-intensity on T2WI. CWs were also present in the lumen of superior sagittal sinus. Arachnoid granulation in the lumen **(A)**, lateral sinus **(B)**, and subarachnoid space **(C)**. Both longitudinal lamellae **(D)** and trabeculae **(E)** in the lumen of superior sagittal sinus. Graphs showing comparisons of the number of type I, type II and type III in the superior sagittal sinus **(F)**.

With the thin layer MRI scanning, three types of AG in SSS were delineated from normal cerebral tissues with hyper-intensity on T_2_WI (**Figures 3A–C**). Longitudinal lamellae and trabeculae were also observed in SSS (**Figures 3D–E**). There were 365 counts of AGs in examined sinuses, averaging 1.36 + 2.58 per SSS. The percent of Types I, II and III was 20.27%, 45.20%, and 34.52% respectively in 268 patients (**Figure 3F** and **Table 1**). There was no difference in the types of AG between female patients and male patients (p=0.352)

### Chordae Willisii Around the Arachnoid Granulation

#### Endoscopic Observations

**Figure 4:**
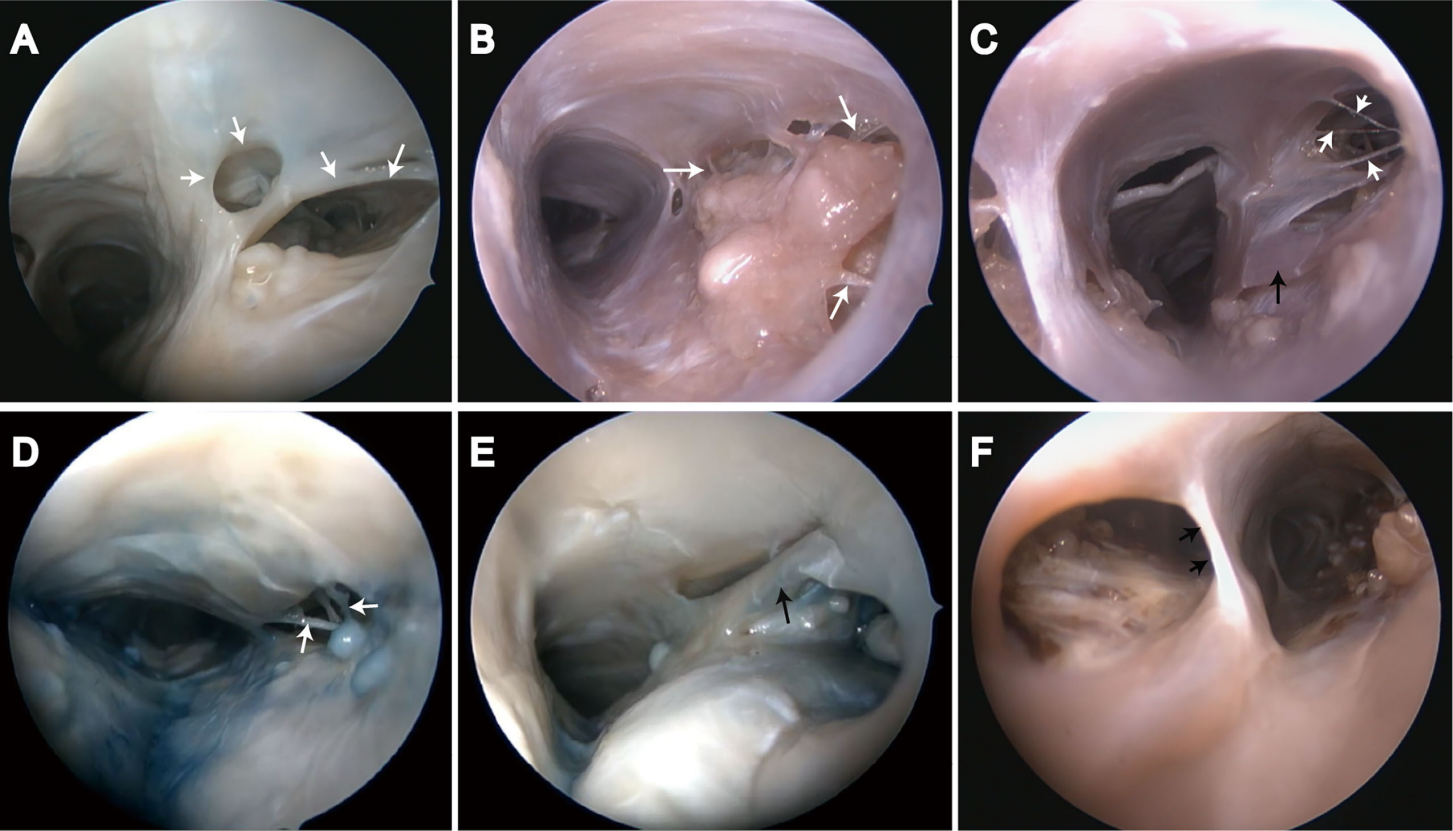
Endoscopic view of chordae willisii around arachnoid granulation in the superior sagittal sinus. **(A)** Valvelike chordae (white arrow); **(B–D)** Trabecular chordae (white arrow); **(E, F)** Longitudinal lamellae (black arrow).

Various sized chambers were formed by valve-like lamellae, lateral walls, and upper wall in the SSS. Door-like structures were developed by valve-like lamellae, and AGs were located in chambers (**Figure 4A**). The trabeculae could be found either in or outside the chambers and appeared either solitary or in clusters (**Figures 4B–D**). Laminar chordae were also observed around the arachnoid granules, and arachnoid granules were fixed to the sinus wall (**Figures 4E, F**).

#### Morphological Observations

**Figure 5:**
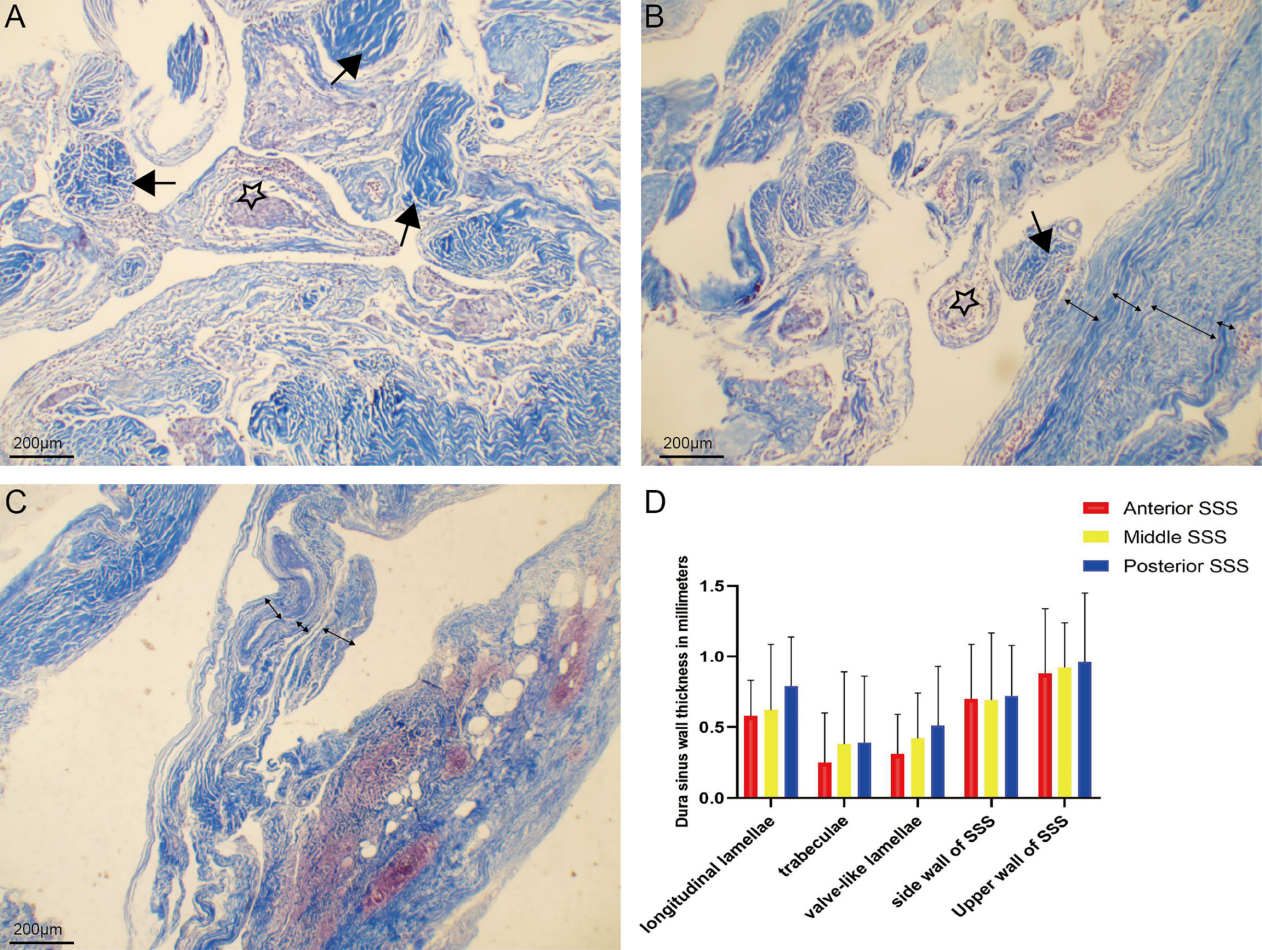
Chordae willisiis around arachnoid granulation were arranged irregularly **(A)**. Chordae willisiis revealed the presence of one layer **(B)**, and dura sinus wall with multiple layers **(C)**. Comparison of thickness of trabecular chordae, longitudinal lamellae, valve-like lamellae and dural sinus wall in the superior sagittal sinus **(D)**.

Chordae willisii around AGs were arranged irregularly. The presence of one layer was revealed with microscopic studies of CW transverse sections and dura sinus wall with multiple layers (**Figures 5A–C**). The thickness of longitudinal lamellae around the AG was the same as the side walls of the SSS and greater than trabeculae or Valve-like lamellae (**Table 1** and **Figure 5D**).

## Discussion

This study demonstrated that arachnoid granulations (AGs) were located on the surface of the sinus wall, in the lateral sinus cavity, or the subarachnoid space. Collagen fibers around AGs were disorderly arranged, and CWs around AGs revealed the presence of multiple layers. The anatomical and histological characteristics of AGs could result in different parasagittal meningioma growth patterns.

### Structure of AGs

AGs were pseudopodia anatomic structures that protrude into the venous sinuses lumen. AGs were detected by an anatomy and MRI scan. Some studies stated the presence of intrasinus structure in the SSS with the aid of a rigid endoscopy. They found arachnoid granulation protruded from the venous lacuna into the lumen of the SSS. With age, the percentage of patients with AGs in SSS increases significantly and there are no AGs in the dura sinuses regarding numerous individuals of various ages (14). The AGs in the cranial bones were discovered for the first time around the SSS at the age of 10, and their number grows dramatically with time. AGs were more prevalent in the cranial bones than in dura sinuses after the age of 60. Three-dimensional high-resolution magnetic resonance imaging sequences such as T2-weighted sampling perfection with application optimized contrasts using different flip-angle evolution and post-contrast T1-weighted magnetization prepared rapid gradient echo was used to diagnose AGs in our study (21). AGs could be clearly observed by an endoscopy and are divided into three types based on their anatomical position. The three types of AGs were also confirmed on the MRI of normal population.

Many studies focused on the histological characteristics of AGs. AGs were made of four distinct components: a central core, a cap cell cluster, an arachnoid cell layer, and a fibrous capsule (22). The arachnoid cell layer that encircled the central core was mostly covered by a thin fibrous capsule with an endothelial investment. The arachnoid cell layer was thickened in places, forming cap cell clusters (23). The central core is contained by arachnoid cells network mixed with connective tissue fibers. Vimentin was found to be localized to intermediate filaments as determined by ultrastructural immunohistochemistry. Depending on their location, the arachnoid cells showed a marked variety in both the cell forms and the number of intermediate filaments or desmosomes. The ultrastructure of arachnoid cell membranes was also investigated by a conventional transmission electron microscope in human AGs. Arachnoid cells exhibited extensive membrane in granulations, namely, desmosomes, gap junctions, tight junctions, and intermediate junctions (9). The arachnoid cells in AGs are not only densely adherent to form a firm structure for CSF transit, but the arachnoid cells also lining the CSF channel exhibit intensive cell–cell contact (24, 25). Similar to previous studies, AGs refer to a narrow neck, broad body, and wide bottom in the dura. The bottom portion protruding into the dura mater formed a single or finger-like dural sheath. We found that type I AGs were larger than type II AGs and the arrangement of collagen fibers in the bottom of type I was more disordered than that of type III. Furthermore, the arachnoid cells were evenly distributed in the body, bottom, and neck.

### Chordae Willisii Around the Arachnoid Granulation

The morphological characteristics of CW in the SSS resulted in the classification of CW into three distinct forms: lamellae resembling valves, longitudinal lamellae, and trabeculae. The most prevalent form was valve-like lamellae, whereas the longitudinal lamellae were the least common form (26). CMs were visualized and described with the aid of a rigid endoscopy. They also identified three types of CW in all examined specimens (27, 28). Similar to previous research, they also confirmed that CW was the most common in the parietooccipital region of the SSS and its most common type was the valve-like. The relationship between CW and dura sinus walls was demonstrated, and CW divided the lumen of the dura sinus into two separate parts. The thickness of CWs was variable in different parts of dural sinuses (29). In our study, we paid more attention to CW around AGs. We found that valve-like lamellae were presented in type II AG, trabeculae in type I AG, and longitudinal lamellae in type III AG. The collagen fibers on the sinus wall were loosely arranged in type I AG.

### Clinical Significance

**Figure 6:**
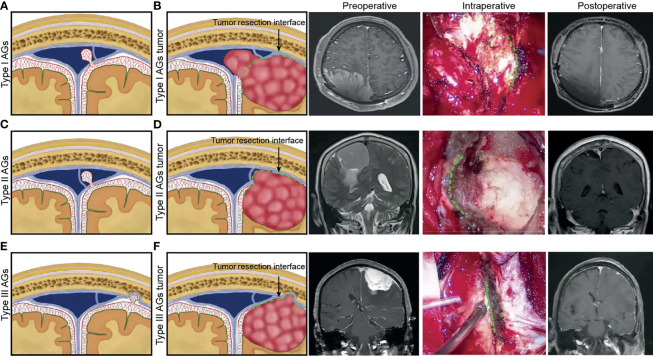
For different anatomical type of arachnoid granulation, schematic diagram illustrating the hypothesized different growth patterns of parasagittal meningioma was presented. Preoperative, intraoperative and postoperative pictures of different types of meningioma were used to illustrate the type of tumor origin and the interface for maximum safe resection of tumors during operation. **(A)** Type I AGs. **(B)** tumor growth patterns of Type I AGs and tumor resection interface (green dotted line) during surgery. **(C)** Type II AGs. **(D)** tumor growth patterns of Type II AGs and tumor resection interface (green dotted line). **(E)** Type III AGs. **(F)** tumor growth patterns of Type III AGs and tumor resection interface (green dotted line).

Parasagittal meningioma grew inside the dural sinus and may displace or conform to the CW with lumen occlusion without expanding through it (30). The chordae may thus provide a barrier to its spreading into adjacent dura sinus. If the tumor enlarges and extends through CW well behind, the chordae may complicate the process by acting as a barrier to getting behind it and entirely removing the tumor. The dura sinus wall incision, which is often limited to the area where the tumor infiltrates the wall, does not have to provide appropriate exposure, making it necessary for extension behind CW (29). Based on anatomical and histological characteristics of AGs and CWs, we summarize the different growth patterns of PMS (**Figure 6**). The tumors originating from type I AGs grew inside or outside the SSS lateral wall, and tumors outside the sinus wall could completely achieve tumor resection. To ensure venous blood flow in the SSS, tumor protruding into sinus was partially removed. Residual tumor was treated with radiotherapy three months after operation and observed by imaging. The tumor originating from type II AGs grew into the lateral sinus and subdural space. For CWs blocked tumor growth into the sinus lumen, resectioning the tumor on the outside of CW could safely and completely achieve maximum tumor resection. The tumors originating from type III AGs grew into subdural space. The dura mater, which was invaded by the tumor, could also be resected completely.

### Limitations

We recognize that our study has a number of limitations. First, cadaveric heads vascular replica did not perfectly reflect the flexibility of intracranial vessels. Second, it makes no recommendations for avoiding intraoperative damage to CWs during tumor removal. Third, it does not identify which part of the tumor invaded the dura mater.

### Conclusion

This study uses anatomical and histological techniques to reveal the different anatomical types of AGs. Meanwhile, the morphological structure of CWs around AGs was described. Based on the anatomic characteristics of AG, we speculate the different growth patterns of PMS, which guided the surgeon to remove the tumor safely.

## Data Availability Statement

The original contributions presented in the study are included in the article/supplementary material. Further inquiries can be directed to the corresponding authors.

## Ethics Statement

The studies involving human participants were reviewed and approved by the Ethics Committee of Guangxi Medical University. The patients/participants provided their written informed consent to participate in this study.

## Author Contributions

Conception and design: YY and WG. Acquisition of data: YY. Analysis and interpretation of data: YY, JG, and MQ. Drafting the article: YY, JD, and LL. Statistical analysis: WX. Study supervision: JD. All authors listed have made a substantial, direct, and intellectual contribution to the work and approved it for publication.

## Funding

This work was supported by the Clinical Research of Liuzhou [grant no. 2021CBB0103], the Clinical Research of Guangxi Autonomous Region (grant no. Z20210107), the Clinical Research of Guangxi Autonomous Region (grant no. Z20200158), the Clinical Research of Liuzhou General Hospital [grant no. LRYGCC202120], the Liuzhou Clinical Research Support Project [2018AF10502] (to YY), the Research Foundation for Advanced Talents of Guizhou Medical University (grant no. University Contract of Doctors J [2021] 014), and the Natural Science Foundation of Guizhou Medical University Incubation Program (grant no. 20NSP084) to JD.

## Conflict of Interest

The authors declare that the research was conducted in the absence of any commercial or financial relationships that could be construed as a potential conflict of interest.

## Publisher’s Note

All claims expressed in this article are solely those of the authors and do not necessarily represent those of their affiliated organizations, or those of the publisher, the editors and the reviewers. Any product that may be evaluated in this article, or claim that may be made by its manufacturer, is not guaranteed or endorsed by the publisher.

## Abbreviations

PSM, parasagittal meningioma; SSS, superior sagittal sinus; AG, arachnoid granulation; CW, chordae willisii.
